# miR-200c Accelerates Hepatic Stellate Cell-Induced Liver Fibrosis via Targeting the FOG2/PI3K Pathway

**DOI:** 10.1155/2017/2670658

**Published:** 2017-06-13

**Authors:** Tengfei Ma, Xiuqin Cai, Zifeng Wang, Li Huang, Chang Wang, Songshan Jiang, Yunpeng Hua, Quentin Liu

**Affiliations:** ^1^Department of Liver Surgery, The First Affiliated Hospital, Sun Yat-sen University, Guangzhou 510080, China; ^2^Sun Yat-sen University Cancer Center, State Key Laboratory of Oncology in South China, Collaborative Innovation Center of Cancer Medicine, Guangzhou, China; ^3^Institute of Cancer Stem Cell, Dalian Medical University, Dalian, China; ^4^Department of Biliary and Pancreatic Surgery, The First Affiliated Hospital, Sun Yat-sen University, Guangzhou 510080, China; ^5^School of Life Sciences, Sun Yat-sen University, Guangzhou 510080, China

## Abstract

**Background:**

Although expression of miR-200s is aberrant in liver fibrosis, its role in liver fibrogenesis still remains unknown. Here, we investigated the role of miR-200c in the activation of human hepatic stellate cells (HSCs) and induction of liver fibrosis.

**Methods:**

We engineered human HSCs (LX2 cell line) to stably express miR-200c (LX2-200c) or empty vector control (LX2-nc).

**Results:**

miR-200c expression upregulated *α*-smooth muscle actin (SMA) and vimentin, enhanced HSCs growth and migration, increased expression of collagen type I (a main component of ECM) gene and secretion of epidermal growth factor (EGF), and upregulated the phosphorylation of Akt, a downstream effector of the PI3K pathway. As a target of miR-200s and inhibitor of PI3K pathway, FOG2 protein expression was significantly suppressed in LX2-200c cells. Moreover, LY294002, a highly selective inhibitor of PI3K, blocked phosphorylation of Akt and the effects of miR-200c.

**Conclusions:**

These data suggest that miR-200c activates HSCs in liver fibrosis possibly by downregulating FOG2 protein expression and upregulating PI3K/Akt signaling. Autocrine activation of EGF signaling may also be a mechanism of miR-200c-mediated HSCs activation. So miR-200c can be a potential marker for HSCs activation and liver fibrosis progression, as well as a potential target to attenuate liver fibrosis.

## 1. Introduction

Liver fibrosis is a common outcome of chronic hepatic injuries including viral hepatitis, alcoholic or nonalcoholic steatohepatitis, autoimmune and chronic inflammatory conditions, and metabolic disorders. It usually progresses toward cirrhosis and eventually induces liver failure and hepatocellular carcinoma (HCC), which is recognized as a source of increasing morbidity and mortality worldwide. The prevalence of cirrhosis has been estimated at 0.3% in the United States and Western Europe and 1-2% globally. It is the 14th leading cause of mortality globally, 12th leading cause of mortality in the United States, and the 4th leading cause in Central Europe and Mexico [1–3]. However, there are few effective therapies for liver fibrosis because its mechanisms are still complicated and poorly understood.

It is well-known that liver fibrosis is an excessive wound healing response to most forms of chronic liver disease and is characteristic of the excessive deposit of extracellular matrix (ECM) proteins. Hepatic stellate cells (HSCs) are recognized as the main matrix-producing cells in the liver. Following a fibrogenic stimulus, the HSCs change from quiescent vitamin A-storing cells to activated myofibroblast-like cells with ECM increasing dramatically, especially type I collagen. Besides secreting ECM, activated HSCs also produce a number of profibrotic cytokines and growth factors to both maintain and deteriorate the fibrotic process in paracrine and autocrine ways [4–9]. So HSCs may be a good target for prevention and treatment of liver fibrosis.

MicroRNAs (miRNAs) are endogenous small noncoding RNA sequences of 20–23 nucleotides that control gene expression by degrading target mRNAs or suppressing their translation. Over one-third of human genes appear to be the targets of conserved miRNAs, which are thought to be involved in various biological processes (normal growth and development) and pathological processes (cancer and other diseases). However, evidence is accumulating that miRNAs participate in the progression of liver fibrosis and regulation of HSCs proliferation/apoptosis [10–12]. The miR-200s (miR-200a, miR-200b, miR-200c, miR-429, and miR-141) have recently been implicated in tissue fibrosis [13, 14]. For example, expression of miR-200s was elevated in a mouse unilateral ureteral obstruction (UUO) model and the expression of miR-200a and -200b was increased in a mouse liver fibrosis model and human clinical samples. Feng et al. [15] also found upregulation of miR-200a, 200b, and 200c in fatty liver tissues. Moreover, upregulation of miR-200b stimulated proliferation and migration of HSCs via the PI3K/Akt signaling pathway [16], while upregulation of miR-200a inhibited TGF-*β*1-induced HSC activation and proliferation [17]. However, few reports have addressed the role of miR-200c in HSC activation and liver fibrosis. Our results in the present study revealed that miR-200c plays a critical role in HSC activation and liver fibrosis progression via targeting FOG2 and activating PI3K/Akt signaling.

## 2. Material and Methods

### 2.1. Reagents and Materials

The human hepatic stellate cell line (LX-2) and the human embryonic kidney cell line cells (HEK 293T) were obtained from American Type Culture Collection (ATCC). Dulbecco's modified Eagle's medium (DMEM) was purchased from Invitrogen Life Technologies, Inc. (Carlsbad, CA, USA). Fetal bovine serum (FBS) and Opti-MEM were obtained from GIBCO (Los Angeles, CA, USA). Anti-Akt, anti-phospho-Akt, anti-GAPDH antibodies, and LY 294002 (PI3K inhibitor) were obtained from Cell Signaling Technology Inc. (Danvers, MA, USA). Anti-FOG2 and anti-*α*-smooth muscle actin (SMA) antibodies were purchased from Abcam (New Territories, Hong Kong). Anti-vimentin antibody was obtained from Millipore (Schwalbach am Taunus, Germany). Lipofectamine 2000 and Trizol were obtained from Invitrogen. Cell Counting Kit-8 (CCK-8) was from Dojindo (Kumamoto, Japan). miExpress™ Precursor miRNA Expression (pEZX-MR03) clone was ordered from GeneCopoeia (Rockville, MD, USA). Universal-RT-microRNA-PCR kits were from EXIQON (Vedbaek, Denmark). SYBR Green qPCR superMix was from Transgen Biotech (Beijing, China).

### 2.2. Cell Culture and Construction of the HSCs Stably Overexpressing miR-200c

The LX-2 cells and 293T cells were cultured in DMEM supplemented with 10% FBS, 100 U/mL penicillin, and 100 *μ*g/mL streptomycin at 37°C in a humidified atmosphere of 5% CO_2_. Briefly, we first generated miR-200c-eGFP lentiviral particles or control scramble lentiviral particles. The 293T cells were cotransfected with 5 *μ*g of either hsa-miR-200c-eGFP carrying plasmid (Catalog No.: HmiR0180-MR03, GeneCopoeia) or 5 *μ*g of pEZX-MR03 (Catalog No.: CmiR0001-MR03, GeneCopoeia) and second generation packaging plasmid (7.5 *μ*g of psPAX2) and envelop plasmid (2.5 *μ*g of pMD2G). The virus-containing supernatant was collected 48 and 72 h after transfection and passed through a 0.5 *μ*m sterile filter. Then, 2 × 10^4^ LX2 cells were infected in a 35 mm dish by 1 ml of miR-200c-expressing or scramble lentiviruses containing 1 *μ*l of polybrene (8 mg/ml). Three days after transduction, the cells were trypsinized and replated in 60-mm dishes. Puromycin (2 *μ*g/ml) was added 3 days after transfection to select and purify the miR-200c-expressing or scramble clones.

### 2.3. Real Time qPCR

RNA was extracted using Trizol (Invitrogen) according to the manufacturer's instructions. cDNA was synthesized from 2 *μ*g of total RNA using a high-capacity cDNA Reverse Transcription Kit (Invitrogen) for mRNA and Universal cDNA synthesis kit II (EXIQON) for miRNA, respectively. Oligo dT primers were used for reverse transcription of mRNA, whereas stem-loop RT primers were employed for reverse transcription of miRNA. Real time quantitative PCR (qPCR) for miRNA expression analysis was performed on a LightCycler 480 Instrument II (Roche Life Sciences, Penzberg, Germany) using the Universal-RT-microRNA-PCR Kit (EXIQON). qPCR for mRNA expression analysis was performed on an Bio-Rad CFX96 Real Time PCR System (Bio-Rad, Pleasanton, CA, USA) using the SYBR Green method (Invitrogen). GAPDH served as the control for mRNA analyses, and U6 snRNA served as the control for miRNA analyses. Primer sequences and/or ordering information used for qPCR analyses are displayed in Table 1. The relative expression levels of miRNAs and mRNAs were calculated by the 2-ddCt method.

### 2.4. Western Blot Analysis

Protein extraction and immunoblot analysis were performed as described previously [18]. Briefly, cells were washed three times with ice-cold PBS and lysed in radioimmunoprecipitation assay (RIPA) lysis buffer (0.01 mol/L sodium phosphate, pH7.2, 150 mmol/L NaCl, 2 mmol/L EDTA, 1% NP-40, 1% sodium deoxycholate, 0.1% SDS, 2 mmol/L AEBSF, 130 mmol/L bestatin, 14 mmol/L E-64, 0.3 mmol/L aprotinin, and 1 mmol/L leupeptin). Total cell lysates were resolved on denaturing and reducing 10–12% SDS-PAGE, and the proteins were transferred from the gel onto Immobilon-P membranes. The membrane was blocked with 5% bovine serum albumin (BSA) in PBS, incubated with different antibodies, incubated with horseradish peroxidase- (HRP-) conjugated secondary antibody, and treated with clarity enhanced chemiluminescence (ECL) substrate (Millipore) to visualize the protein bands.

### 2.5. Proliferative Analysis

LX2-200c and LX2-nc cells were seeded at 1 × 10^3^ cells/well in 96-well plates, respectively, with or without treatment of LY294002, cultured for 0, 1, 2, 3, 4, and 5 days, incubated with 10 *μ*L of CCK-8 plus 100 *μ*L of DMEM/well for 2 h, and then placed in a microplate reader (BioTek Synergy2, Winooski, VT, USA) to measure the absorbance at 450 nm.

### 2.6. Wound Healing Assay

LX2-200c and LX2-nc cells were seeded at 5 × 10^4^ cells/well in 6-well plates, respectively, with or without treatment of LY294002. When cells were grown to 80% confluence, a wound track was scraped using a sterile 200-*μ*l pipette tip to create a denuded zone of constant width in the middle of each well. Then, each well was washed with PBS twice to remove the detached cells. Cell migration to the wounded region was observed under a microscope. Images were captured at 0, 12, and 24 h to monitor the wound healing process. Migration ratio (%) was calculated by dividing the width of the denuded zone at 12 h or 24 h by its width at 0 h.

### 2.7. ELISA

The conditioned medium was collected. Briefly, LX2-200c and LX2-nc cells were seeded at 5 × 10^4^ cells/well in 6-well plates, respectively, grown to 80% confluence, and washed with sterile PBS three times. The growth medium was replaced with an equal volume of serum-free DMEM and, after 24 h, the supernatant was centrifuged to remove cells and particulates, collected, and stored at −80°C.

Human EGF protein levels were quantified by ELISA using the colorimetric sandwich ELISA kit (Proteintech, Chicago, ILUSA) according to the instruction manual. Briefly, samples and standards were diluted with PT 1-ec diluent in wells precoated with a monoclonal anti-human EGF antibody and treated with 1% BSA in PBS for 1 h to block nonspecific binding. The plates were incubated for 2 h at room temperature to allow capture of the EGF-tagged protein by the bound antibody, extensively washed with buffer, incubated with a biotinylated polyclonal antibody to EGF for 1 h at 37°C in a humidified environment, washed with buffer, incubated with Streptavidin-HRP for 40 min, washed, incubated with TMB (tetramethylbenzidine) substrate for 10 min, and incubated with stop solution to terminate the color reaction. Absorbance was measured at 450 nm.

### 2.8. Statistical Analysis

Each experiment was performed in at least triplicate. Results are expressed as the mean ± standard deviation. Data were analyzed using GraphPad Prism (GraphPad Software Inc., La Jolla, CA). Statistical analysis was performed using the Student* t*-test. *P* < 0.05 was considered statistically significant.

## 3. Results

### 3.1. miR-200c Promotes the Activity, Proliferation, and Migration of HSCs

**Figure 1:**
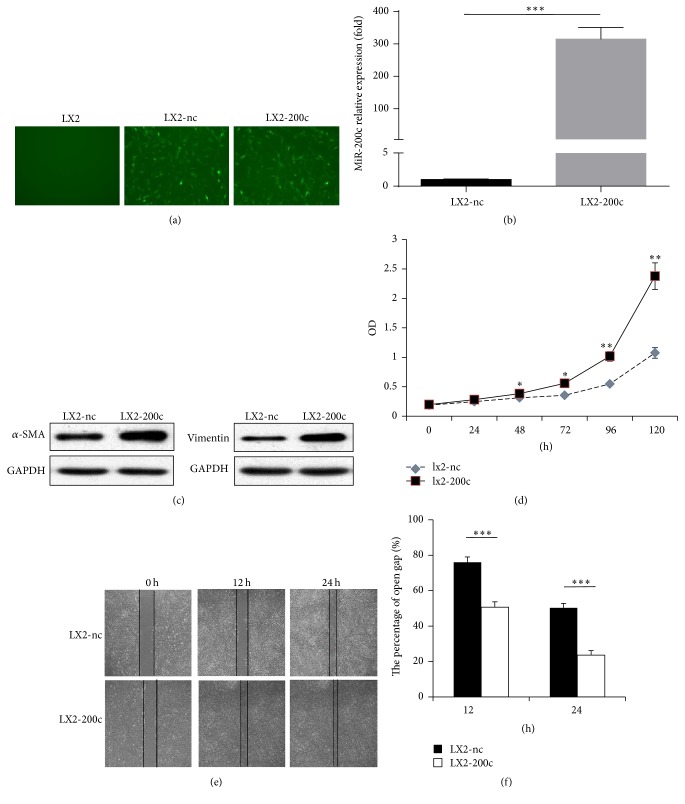
miR-200c promotes the activity, proliferation, and migration of HSCs. (a, b) Construction of two cell lines (LX2-200c and LX2-nc) from the human HSC line (LX2). LX2-200c stably overexpressed miR-200c and LX2-nc expressed empty vector as the control. Green fluorescent protein (GFP) expression was observed in LX2-200c and LX2-nc by fluorescence microscopy, rather than in LX2 (original magnification ×400). The level of miR-200c expression in LX2-200c was approximately 314 times of that in LX2-nc cells. (c) LX2-200c had markedly higher levels of *α*-SMA and vimentin. (d**)** miR-200c promoted the proliferation of LX2. (e, f) The migration of LX2 was increased by miR-200c. *∗* means *p* < 0.05, *∗∗* means *p* < 0.01, and *∗∗∗* means *p* < 0.001.

First, we constructed two cell lines from the human HSC line (LX2): one stably overexpressed miR-200c (LX2-200c) and its control that expressed empty vector (LX2-nc) (*P* = 0.013, Figures 1(a) and 1(b)). We found that LX2-200c had markedly higher levels of *α*-SMA and vimentin (classical activation markers of HSCs [19]; Figure 1(c)) and significantly higher cell growth rate (all *P* < 0.05; Figure 1(d), and Table 1). The wound healing assay showed that more LX2-200c cells than LX-2-nc cells migrated into the wound track after 12 h and 24 h. The wound track area after 12 h and 24 h was markedly smaller for LX2-200c than for LX2-nc (50.7%  ± 3.1% and 23.7%  ± 2.5%, respectively, versus 76.0%  ± 3.0% and 50.3%  ± 2.5%, respectively; all *P* < 0.001; Figures 1(e) and 1(f)).

### 3.2. miR-200c Activates the PI3K/Akt Pathway via Suppressing Expression of FOG2

**Figure 2:**
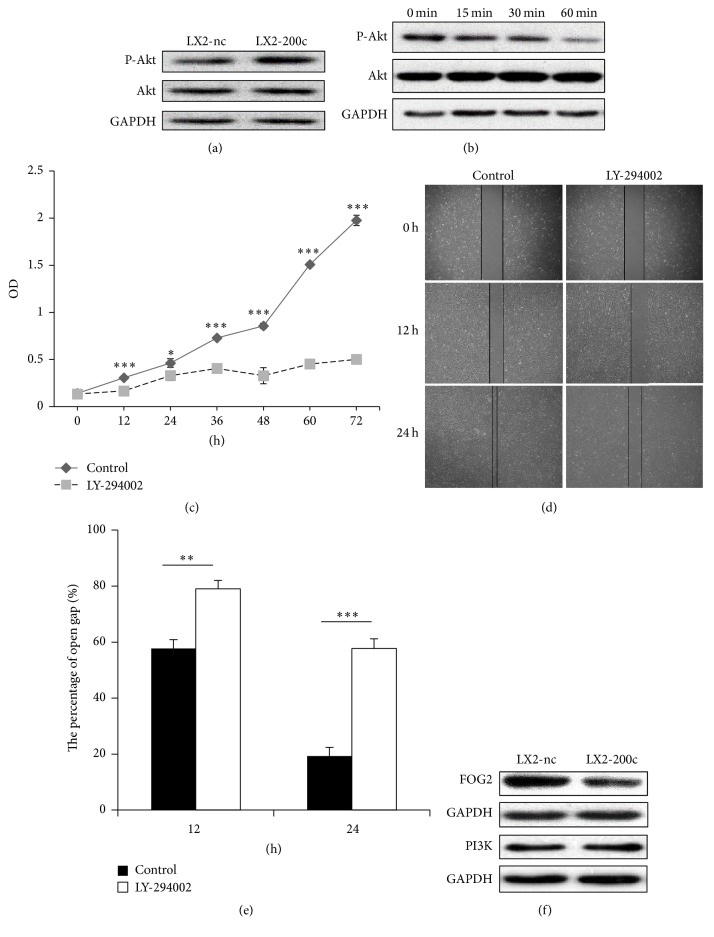
miR-200c activates the PI3K/Akt pathway via suppressing expression of FOG2. (a) The level of phosphorylation at Akt residue S473 was significantly higher in LX2-200c cells than LX2-nc cells. (b) 25 *μ*M LY-294002 (a PI3K inhibitor) significantly inhibited the expression of phospho-Akt in LX2-200c cells in a time-dependent manner. (c–e) 25 *μ*M LY-294002 significantly inhibited the proliferation and migration of LX2-200c cells. (f) There was the significantly lower levels of FOG2 expression in LX2-200c cells, while no significant change was observed in PI3K expression. *∗* means *p* < 0.05, *∗∗* means *p* < 0.01, and *∗∗∗* means *p* < 0.001.

Because activation of the PI3K/Akt pathway is required for HSC proliferation and migration [20], we further probed whether the proliferative and migratory effects of miR-200c are dependent on the activation of PI3K/Akt. Our results showed that the level of phosphorylation at Akt residue S473 was significantly higher in LX2-200c cells than LX2-nc cells (Figure 2(a)). We also observed that 25 *μ*M LY-294002 (a PI3K inhibitor) significantly inhibited the expression of phospho-Akt in LX2-200c cells in a time-dependent manner (Figure 2(b)) and blocked the proliferation and migration of LX2-200c cells (Figures 2(c) and 2(d)). Moreover, the wound area for LX2-200c cells after 12 h and 24 h was significantly larger with LY-294002 treatment than without treatment (79%  ± 3.0% and 57.7%  ± 3.5%, respectively, versus 57.7%  ± 3.2% and 19.3%  ± 3.1%, respectively; all *P* < 0.001; Figures 2(d) and 2(e)).

To further probe the mechanism of miR-200c-mediated PI3K signaling, we measured the expression of FOG2 (a reported inhibitor of PI3K [16, 21–23]) and PI3K after miR-200c transfection and found that LX2-200c cells had significantly decreased levels of FOG2 while no significant change was observed in PI3K expression (Figure 2(f)), which is consistent with our previous finding of increased Akt phosphorylation.

### 3.3. miR-200c Overexpression Upregulates the Expression of Collagen Type I and Epidermal Growth Factor (EGF) via the FOG2/PI3K/Akt Pathway

**Figure 3:**
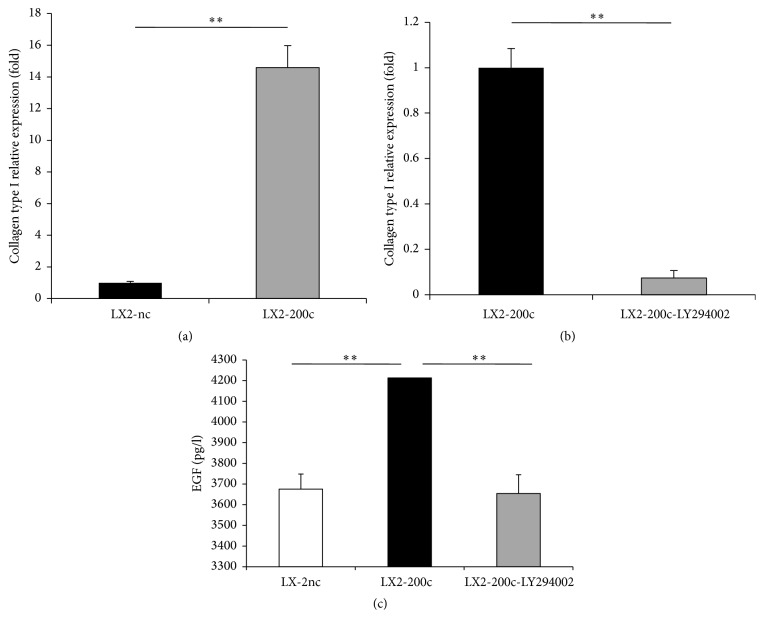
miR-200c overexpression upregulates the expression of collagen type I and epidermal growth factor (EGF) via the FOG2/PI3K/Akt pathway. (a, c) miR-200c overexpression significantly upregulated the gene expression of collagen type I and EGF secretion in HSCs. (b, c) 25 *μ*M LY-294002 (a PI3K inhibitor) significantly inhibited the gene expression of collagen type I and EGF secretion in LX2-200c cells. *∗∗* means *p* < 0.01.

Collagen type I is the main component of the ECM and dramatically increases in liver fibrosis [20]. We found that miR-200c overexpression significantly upregulated the gene expression of collagen type I in HSCs (Figure 3(a)). To assess whether PI3K/Akt signaling is involved in miR-200c-mediated collagen type I gene expression, we treated LX2-200c cells with 25 *μ*M LY294002 in the presence of 10% serum for 30 min and detected a marked drop in their collagen type I mRNA levels (Figure 3(b)).

Epidermal growth factor (EGF) plays a role in liver fibrosis through promoting the autocrine/paracrine proliferation and activation of HSCs [24–26]. Assessment of EGF protein level in the conditioned medium of HSCs revealed a significantly increased level of EGF secretion by HSCs after miR-200c transfection (Figure 3(c)), which could be inhibited by treatment with 25 *μ*M LY294002 for 30 min (Figure 3(c)). These findings proved that PI3K/Akt signaling is involved in miR-200c-enhanced secretion of EGF.

## 4. Conclusion

Hepatic stellate cells (HSCs) are thought to play a crucial role in liver fibrosis, as their activation following liver injury is responsible for increased synthesis and deposition of ECM proteins in the liver. In addition, the proliferation and migration of HSCs after activation effectively amplify the fibrotic response. Furthermore, profibrotic cytokines and growth factors secreted by activated HSCs (like transforming growth factor-*β* [TGF-*β*], platelet-derived growth factor [PDGF], matrix metalloproteinases [MMPs], epidermal growth factor [EGF], leptin, and so on [4–9]) perpetuate the fibrotic process through paracrine and autocrine effects.

Although several studies have reported the involvement of miR-200s in the development of tissue fibrosis, including liver fibrosis, no study has implicated miR-200c. Additionally, miR-200a and miR-200b were found to have opposite effects on liver fibrosis development [13–17]. Herein, we showed that proliferation and migration of a human HSC (LX-2) cell line were enhanced by engineering it to stably overexpress miR-200c (LX2-200c). The expressions of *α*-SMA and vimentin (biomarkers of activated HSCs) and collagen type I (a major component of ECM) were found to be upregulated significantly in LX2-200c.

The phosphatidylinositol 3-kinase (PI3K)-Akt pathway plays a key role in cellular hypertrophy. The PI3K enzyme, a well-known upstream mediator of Akt kinase activation, is composed of a catalytic subunit, p110, and a regulatory subunit, p85*α*. The catalytic subunit of PI3K can recruit Akt kinase to the membrane and activate it by phosphorylation. Activated Akt further phosphorylates several downstream proteins that play central roles in hypertrophy, cell growth, cell survival, and protein synthesis [20]. However, a novel protein FOG2 binds to p85*α*, thereby inhibiting PI3K activation. Intriguingly, miR-200 was reported to decrease FOG2 expression by targeting the 3′ UTR of the FOG2 mRNA, thereby altering PI3K activity and regulating the insulin signaling pathway and metabolism [16, 21]. Park et al. [22] indicated that FOG2 is downregulated by mimics of miR-200b and miR-200c in mouse mesangial cells (MMC). Mei et al. [23] also found that miR-200c inhibited the expression of PTEN and FOG2 to promote the expansion and immune suppressive activity of myeloid-derived suppressor cells (MDSCs). In the present studies, we showed that transfection of miR-200c significantly reduced FOG2 protein levels in LX-2 cells, which subsequently led to PI3K/Akt signaling activation. To determine whether the effect of miR-200c is mediated through the FOG2/PI3K pathway, we used LY294002, a specific PI3 kinase inhibitor, to block PI3K activation in the LX-2 cells transfected with miR-200c. The results showed that LY294002 treatment significantly inhibited miR-200c-enhanced LX-2 cell proliferation and migration and ECM deposition, which suggested that PI3K/Akt activation was essential to the profibrotic effect of miR-200c.

EGF plays a role in liver fibrosis, liver cirrhosis, and even hepatocellular carcinoma (HCC). EGF expression in the liver increases during cirrhosis, and the level of EGF mRNA expression is associated with poor survival of cirrhotic patients. Bachem et al. [24] indicated that EGF signaling triggered HSC proliferation in a receptor-dependent autocrine/paracrine manner. Zhang et al. [25] pointed out that EGF expression was significantly increased in activated HSCs, and EGF promotes HSC proliferation via activation of the EGF receptor (EGFR). Fuch et al. [26] reported that the small-molecule EGFR inhibitor erlotinib inhibited the activation of HSCs, prevented the progression of cirrhosis, and caused fibrosis to regress in some animal models. Our results showed that miR-200c overexpression also promoted the secretion of EGF in HSCs and that miR-200c-mediated EGF secretion could be inhibited by LY294002 treatment. However, our preliminary experiment showed that miR-200c overexpression had no effect on other growth factors, including transforming growth factor-*β*1 (TGF-*β*1), platelet-derived growth factor (PDGF), fibroblast growth factor (FGF), hepatocyte growth factor (HGF), and vascular endothelial growth factor (VEGF). So we speculated that miR-200c promoted HSC proliferation, migration, and ECM production in part via autocrine activation of EGF signaling, rather than signaling by other growth factors. In addition, PI3K/Akt signaling also played a key role in miR-200c-mediated EGF secretion by HSCs.

**Figure 4:**
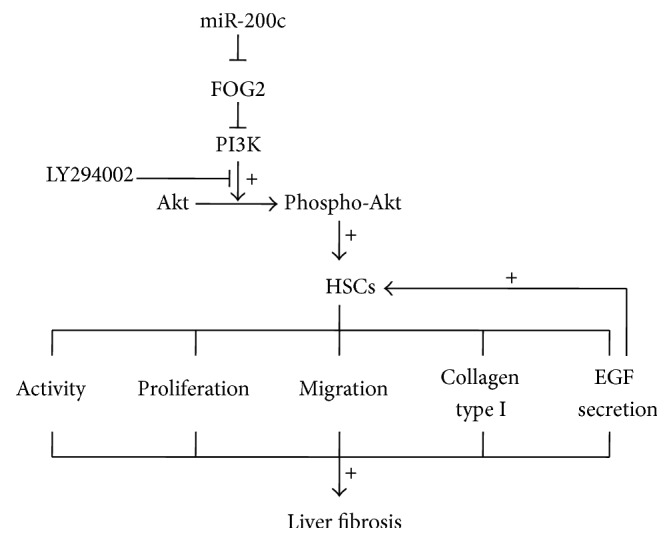
Schematic model of miR-200c accelerating hepatic stellate cell-induced liver fibrosis via targeting the FOG2/PI3K pathway. miR-200c activates HSCs and finally accelerates the progression of liver fibrosis via downregulation of FOG2 protein synthesis, activation of PI3K/Akt signaling. Autocrine activation of EGF signaling may also be a mechanism of miR-200c-mediated HSCs activation.

In summary, our results show directly that miR-200c is an important promoter of HSCs activity, proliferation, and migration and has a very important role in liver fibrosis. We found that the effects of miR-200c on HSCs and liver fibrosis are mediated via downregulation of FOG2 protein synthesis, activation of PI3K/Akt signaling, upregulation of activation of autocrine EGF signaling, and upregulation of collagen type I production in HSCs (Figure 4). In view of its putative pathogenic role in HSCs, miR-200c may be a potential marker for HSC activation and liver fibrosis progression, and even a potential target for the treatment of liver fibrosis.

## Figures and Tables

**Table 1:** Primer sequences and/or ordering information used for qPCR analyses.

Gene	Sense	Antisense
Collagen type I	5′-GTGCGATGACGTGATCTGTGA-3′	5′-TTGGTCGGTGGGTGACTCTG-3′
GAPDH	5′-GGAGCGAGATCCCTCCAAAAT-3′	5′-GGCTGTTGTCATACTTCTCATGG-3′
Hsa-miR-200c	EXIQON, product number: 204482	
U6	EXIQON, product number: 203907	

## Abbreviations

HSCs: Hepatic stellate cells
SMA: *α*-Smooth muscle actin
EGF: Epidermal growth factor
HCC: Hepatocellular cancer
ECM: Extracellular matrix
miRNAs: MicroRNAs
UUO: Unilateral ureteral obstruction
DMEM: Dulbecco's modified Eagle's medium
FBS: Fetal bovine serum
qPCR: Real time quantitative PCR
RIPA: Radioimmunoprecipitation assay
BSA: Bovine serum albumin
ECL: Clarity enhanced chemiluminescence
TGF-*β*: The transforming growth factor *β*
PDGF: Platelet-derived growth factor
MMPs: Matrix metalloproteinases
PI3K: Phosphatidylinositol 3-kinase
MMC: Mouse mesangial cells
MDSCs: Myeloid-derived suppressor cells
EGFR: EGF receptor
FGF: Fibroblast growth factor
HGF: Hepatocyte growth factor
VEGF: Vascular endothelial growth factor.

## Data Access

The datasets during and/or analysed during the current study are available from the corresponding author on reasonable request.
